# Tuberculosis screening in adult patients with inborn errors of immunity: a single-center cohort study

**DOI:** 10.3389/fimmu.2026.1888729

**Published:** 2026-07-24

**Authors:** Pelin Korkmaz, İlkim Deniz Toprak, Merve Hormet Igde, Osman Ozan Yegit, Semra Demir, Nevzat Kahveci, Ayse Feyza Aslan, Mehmet Emin Sezgin, Merve Dilsad Atasever, Şule Çelik Kamacı, Deniz Eyice Karabacak, Bircan Erden, Zeynep Kilinc, Mehmet Sait Yordam, Isil Gogem Imren Aksit, Derya Unal, Aslı Gelincik

**Affiliations:** Department of Internal Medicine, Division of Immunology and Allergy Diseases, Istanbul Universitesi Istanbul Tip Fakultesi, İstanbul, Türkiye

**Keywords:** common variable immunodeficiency disorders (CVID), IEI, inborn errors of immunity, latent TB infections (LTBI), tuberculin skin test (TST)

## Abstract

**Background:**

Data on tuberculosis, a leading cause of infectious disease mortality, in patients with inborn errors of immunity (IEI) are limited.

**Objective:**

This study aimed to evaluate the burden of tuberculosis and the diagnostic utility of the tuberculin skin test (TST) in patients with IEI and to propose a tuberculosis screening strategy.

**Methods:**

Evaluation comprised a comprehensive exposure history, symptom assessment, TST (which was repeated after 1–4 weeks if the initial result was 0–4 mm), and chest imaging including radiography and computed tomography where available. Patients underwent standard clinical assessment for tuberculosis, followed by appropriate treatment and follow-up.

**Results:**

Among 117 patients with IEI [median age = 35 years, interquartile range (IQR) = 26–45.5 years], antibody deficiencies were the most common subgroup (82.1%). All patients had previously received a Bacillus Calmette–Guérin (BCG) vaccination. Of the 105 patients who underwent TST, 19 had a history of tuberculosis, the majority of whom had pulmonary involvement (59.1%), and three had recurrent disease. Three patients diagnosed with pulmonary, gastrointestinal, and lymph node tuberculosis had TST values of 22, 9, and 9 mm, respectively. Two patients with TST measurements of 12 and 6 mm were diagnosed with non-tuberculous mycobacterial lung disease. The median TST was 9 mm (IQR = 1.5–14.25 mm) in patients with tuberculosis and was 4 mm (IQR = 0–10.5 mm) in those without.

**Conclusion:**

This study represents the first comprehensive evaluation of tuberculosis screening and TST assessment in patients with IEI. Pulmonary tuberculosis was the most common site of involvement. In BCG-vaccinated patients with IEI who developed tuberculosis, the TST responses remained below the conventional 15-mm threshold, highlighting the need for revised cutoff values in this population, similar to those recommended for patients with HIV.

## Introduction

Inborn errors of immunity (IEI) are genetic disorders that impair the development or function of the immune system, resulting in heightened susceptibility to various infections, including tuberculosis and non-tuberculous mycobacterial infections (1–3). Certain IEI subtypes, such as Mendelian susceptibility to mycobacterial diseases (MSMD), are particularly associated with mycobacterial infections (4). Chronic granulomatous disease (CGD) and severe combined immunodeficiency (SCID) also increase the risk of tuberculosis. However, the association between antibody deficiencies, including common variable immunodeficiency (CVID), and tuberculosis remains uncertain (5, 6).

Globally, tuberculosis reclaimed its position as the leading cause of mortality attributable to a single infectious pathogen in 2023, having been overtaken by coronavirus disease 2019 (COVID-19) for 3 years. According to the 2024 World Health Organization (WHO) report, an estimated 10.8 million individuals [95% uncertainty interval (UI) = 10.1–11.7 million] contracted tuberculosis in 2023 (7). In the same report, Türkiye continues to be classified among countries that have achieved a 20%–49% reduction in tuberculosis incidence, with an estimated incidence rate of 10–49 cases per 100,000 inhabitants per year (7). Although these figures indicate progress, the target remains an incidence rate of fewer than 10 cases per 100,000 population per year. In 2023, tuberculosis was estimated to have caused approximately 1.25 million deaths globally (95% UI = 1.13–1.37 million), with approximately 1.09 million occurring in HIV-negative individuals and approximately 161,000 among those living with HIV (7).

Latent tuberculosis infection (LTBI) is defined as a persistent immune response to antigens of *Mycobacterium tuberculosis* in the absence of clinically evident active tuberculosis. Although LTBI remains asymptomatic and non-infectious, it is estimated to affect approximately 33% of the global population. Immunocompromised individuals with LTBI are at increased risk of progression to active tuberculosis, particularly within the first 2 years of exposure (8, 9). Therefore, prompt diagnosis and treatment of LTBI are essential to prevent progression to active tuberculosis and to limit its spread, thereby reducing the global burden of tuberculosis (10). Currently, evidence-based data on LTBI in IEI are lacking. The tuberculin skin test (TST) or interferon-gamma release assay (IGRA) may be used to detect LTBI. The TST involves the intradermal administration of a purified protein derivative (PPD), eliciting a delayed-type hypersensitivity response: a positive result is determined by the size of the induration. The dual-TST approach, which addresses the booster phenomenon, improves the accuracy of LTBI detection in high-risk or immunocompromised individuals (11–14). A TST induration of ≥5 mm is considered positive in individuals with HIV infection, whereas an induration of ≥15 mm is the threshold for positivity in the general population (7). However, the TST positivity thresholds for individuals with IEI remain poorly defined. Although guidelines designate an induration of 5 mm or more as positive in certain high-risk groups—such as individuals with HIV infection, recent contacts of persons with active tuberculosis, those exhibiting fibrotic changes on chest radiographs consistent with previous tuberculosis, organ transplant recipients, and immunosuppressed patients, including those undergoing prolonged corticosteroid therapy or receiving tumor necrosis factor alpha (TNF-α) inhibitors—no validated cutoff value has been established for patients with IEI (15, 16). Since these patients may not be adequately represented in existing studies, applying the same criteria to this population poses challenges, highlighting the need for further research in this area. Given their heightened susceptibility to infections, whether routine screening—as recommended for HIV-infected individuals—should be implemented for patients with IEI remains an open question.

Individuals with IEI are considered to be at higher risk of tuberculosis. However, data on its prevalence, clinical distribution, and prevention in this population remain scarce. To address this knowledge gap, the present study aimed to determine the prevalence of previous and active tuberculosis, identify the most common sites of involvement, assess the utility of the TST, and propose a systematic approach to tuberculosis screening in patients with IEI.

## Methods

### Study population and study design

Between 2013 and 2024, a total of 340 adult patients evaluated for suspected IEI at the Allergy and Immunology Outpatient Clinic of Istanbul University, Istanbul Faculty of Medicine, were screened for eligibility. Of these, 223 patients were excluded due to secondary immunodeficiency (*n* = 88), unconfirmed diagnosis (*n* = 17), death prior to enrollment (*n* = 12), pregnancy (*n* = 1), or refusal to participate (*n* = 105). Consequently, 117 adult patients with a confirmed diagnosis of IEI who provided written informed consent were included in the final analysis (**Supplementary Figure 1**). IEI diagnoses were established in accordance with the criteria set by the International Union of Immunological Societies (IUIS) and the European Society for Immunodeficiencies (ESID) (17–20).

### Screening protocol for tuberculosis infection

**Figure 1:**
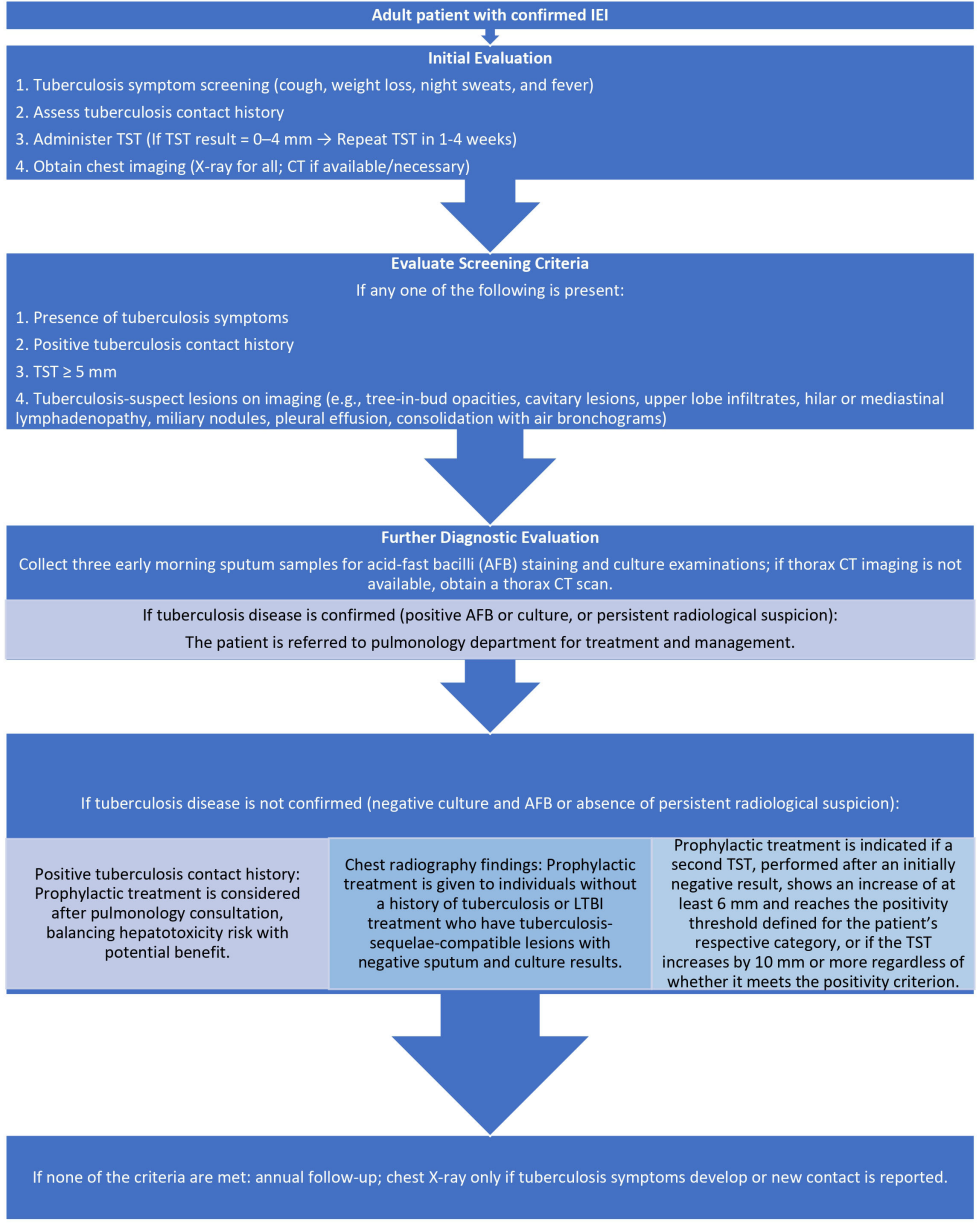
Institutional tuberculosis screening algorithm. TST, Tuberculin skin test; LTBI, Latent tuberculosis infection; CT, Computed tomography.

All patients with a confirmed diagnosis of IEI were evaluated according to our center’s tuberculosis screening protocol at the Adult Allergy and Immunology Outpatient Clinic of Istanbul University, Istanbul Faculty of Medicine, which provides a systematic framework for tuberculosis screening and management (**Figure 1**). As part of our routine clinical assessment, all adult patients diagnosed with IEI undergo TST evaluation both as an *in vivo* measure of cell-mediated immunity and as an integral component of systematic tuberculosis screening. This protocol has been adopted because patients with IEI may be at increased risk of mycobacterial infections and because tuberculosis remains a significant public health concern in Türkiye. The diagnostic assessment and the clinical management of patients with IEI at our center are conducted in accordance with current international guidelines and regional expert recommendations, including the assessment of mycobacterial susceptibility and potential BCG-related complications (21).

Age at evaluation, age at diagnosis, sex, body mass index (BMI), smoking and alcohol use, clinical features, family history, and physical examination findings were systematically documented. Tuberculosis-related information was also obtained, including BCG vaccination status, the presence of a BCG scar, history of tuberculosis, use of antituberculosis therapy, and history of close contact with individuals with active tuberculosis. BCG vaccination status was assessed primarily through patient self-report and verification of a BCG scar during physical examination. Close contact was defined as residing with a tuberculosis patient or having frequent interactions with individuals with the disease. Detailed records were kept on prior tuberculosis infections, including the sites of involvement. Symptoms suggestive of tuberculosis, such as chronic cough, weight loss, night sweats, and fever, were systematically assessed. Given their immunocompromised status, patients were additionally screened for palpable lymphadenopathy and gastrointestinal symptoms, including diarrhea.

The TST was performed by a trained healthcare professional at the tuberculosis dispensary utilizing the Mantoux technique. Specifically, 0.1 ml of PPD was administered intradermally into the volar surface of the left forearm using a tuberculin syringe. The injection site was selected on intact skin to ensure valid test results. The results were evaluated 72 h after administration by the healthcare professional who performed the test. Induration at the injection site was measured in millimeters and interpreted accordingly (9, 22). For individuals with TST results of 0–4 mm, the test was repeated after 1–4 weeks to assess for a booster response.

As part of ongoing clinical monitoring for IEI, many patients had previously undergone computed tomography (CT) imaging—the majority of which was obtained during the COVID-19 pandemic—which was subsequently reassessed by radiologists and pulmonologists. These images were systematically reviewed for post-tuberculosis sequelae, and findings were documented only when they could not be attributed to acute infectious processes, such as COVID-19 pneumonia (23, 24).

Patients presenting with a TST induration of ≥5 mm, tuberculosis-related symptoms, suspicious findings on chest imaging, or a history of close contact with an individual with active tuberculosis underwent additional evaluation for active tuberculosis. Patients meeting at least one screening criterion were evaluated in consultation with a pulmonologist. Further investigations included sputum acid-fast bacillus (AFB) staining and cultures, as well as chest CT scans when indicated. Treatment for LTBI and active tuberculosis was administered according to national guidelines (**Figure 1**) (11).

### Statistical analysis

The Statistical Package for Social Sciences (SPSS) version 27 and Microsoft Excel were utilized for data analysis. The normality of numerical variables was evaluated using the Kolmogorov–Smirnov and Shapiro–Wilk tests. As the data did not conform to a normal distribution, continuous variables were presented as medians and interquartile ranges (IQR, 25th–75th percentile). Spearman’s rank correlation analysis was employed to examine associations between variables. Statistical significance was established at *p*-values less than 0.05.

### Ethical considerations

This study received approval from the Ethics Committee of Istanbul Faculty of Medicine and the Internal Review Board of the Department of Internal Medicine (approval no. 24.06.2024-2602043).

### Human ethics and consent to participate declarations

All procedures conducted in studies involving human participants adhered to the ethical standards set forth by the relevant institutional and/or national research committees, as well as the principles outlined in the 1964 Helsinki Declaration and its subsequent amendments. In addition, written informed consent was secured from each participant involved in the study.

## Results

### Demographic and clinical characteristics and IEI classification

Of the patients, 60 (51.3%) were men, with a median age of 35 years (IQR = 26–45.5). The median disease duration was 6 years (IQR = 3–12.75), and the median age at diagnosis was 30 years (IQR = 17–39). Detailed demographic and clinical data are presented in **Table 1**.

**Table 1:** Demographics and clinical characteristics of the patients.

Characteristic	Patients (n = 117)
Age (years), median (IQR, 25–75)	35 (26–45.5)
Women/men, n (%)	57/60 (48.7/51.3)
Disease duration (years), median (IQR, 25–75)	6 (3–12.75)
Smoker, n (%)	20 (17.1)
Social alcohol consumer, n (%)	11 (9.4)
BMI	
BMI ≤ 18.5, n (%)	12 (12.5)
BMI = 18.6–24.9, n (%)	49 (51)
BMI ≥ 25, n (%)	35 (36.5)
Immunoglobulin replacement therapy	
IVIG, n (%)	28 (23.9)
SCIG, n (%)	31 (26.5)
Prophylactic antibiotics	29 (24.8)
Sulfamethoxazole and trimethoprim combination	17 (14.5)
Azithromycin	5 (4.3)
Amoxicillin and clavulanic acid	5 (4.3)
Sulfamethoxazole and trimethoprim combination, fluconazole and sirolimus	1 (0.9)

IQR, interquartile range; BMI, body mass index; IVIG, intravenous immunoglobulin; SCIG, subcutaneous immunoglobulin.

The most common disease category was antibody deficiencies, accounting for 82.1% of the patients. Patient distribution according to the IUIS classification (18, 19) is presented in **Table 2**. Although genetic testing was available at our center, its use was limited by financial constraints and restricted accessibility. Molecular genetic analysis was conducted when clinically indicated and feasible. In total, 67 of the 117 patients underwent genetic testing, yielding a molecular diagnosis in 20 patients (**Supplementary Table 1**). Whole-exome sequencing was used as the primary genetic testing modality.

**Table 2:** Classification of patients according to the International Union of Immunological Societies (IUIS).

IEI phenotypic classification, n (%)	Patients (n = 117)
1) Immunodeficiencies affecting cellular and humoral immunity	2 (1.7)
Combined immunodeficiency (unspecified)	2 (1.7)
2) Combined immunodeficiencies with associated or syndromic features	7 (6)
DiGeorge/velocardiofacial syndrome, Chr22q11.2 deletion	1 (0.9)
Diseases currently considered as hyper IgE syndromes (HIES)	3 (2.6)
Tricho-hepato-enteric syndrome	1 (0.9)
Ataxia telangiectasia	1 (0.9)
POLE1 (polymerase subunit 1) deficiency (FILS syndrome)	1 (0.9)
3) Predominantly antibody deficiencies	96 (82.1)
Common variable immunodeficiency phenotype	57 (48.7)
Selective IgA deficiency	19 (16.2)
IgG subclass deficiency with IgA deficiency	9 (7.7)
IgG subclass deficiency	3 (2.6)
X-linked agammaglobulinemia	3 (2.6)
Hyper IgM syndromes	4 (3.4)
4) Diseases of immune dysregulation	5 (4.3)
LRBA deficiency	1 (0.9)
CTLA4 deficiency	3 (2.6)
RLTPR (CARMIL2) deficiency	1 (0.9)
Diseases associated with EBV susceptibility	1 (0.9)
5) Congenital defects of phagocyte number or function	4 (3.4)
GATA2 deficiency	1 (0.9)
Chronic granulomatous disease	3 (2.6)
6) Defects in intrinsic and innate immunity	2 (1.7)
WHIM (warts, hypogammaglobulinemia, infections, myelokathexis) syndrome	1 (0.9)
Predisposition to HPV	1 (0.9)
7) Phenocopies of inborn errors of immunity	1 (0.9)
Thymoma with hypogammaglobulinemia (Good syndrome)	1 (0.9)

IEI, inborn errors of immunity; HPV, human papillomavirus; EBV, Epstein–Barr virus.

### Tuberculosis history and disease distribution

**Figure 2:**
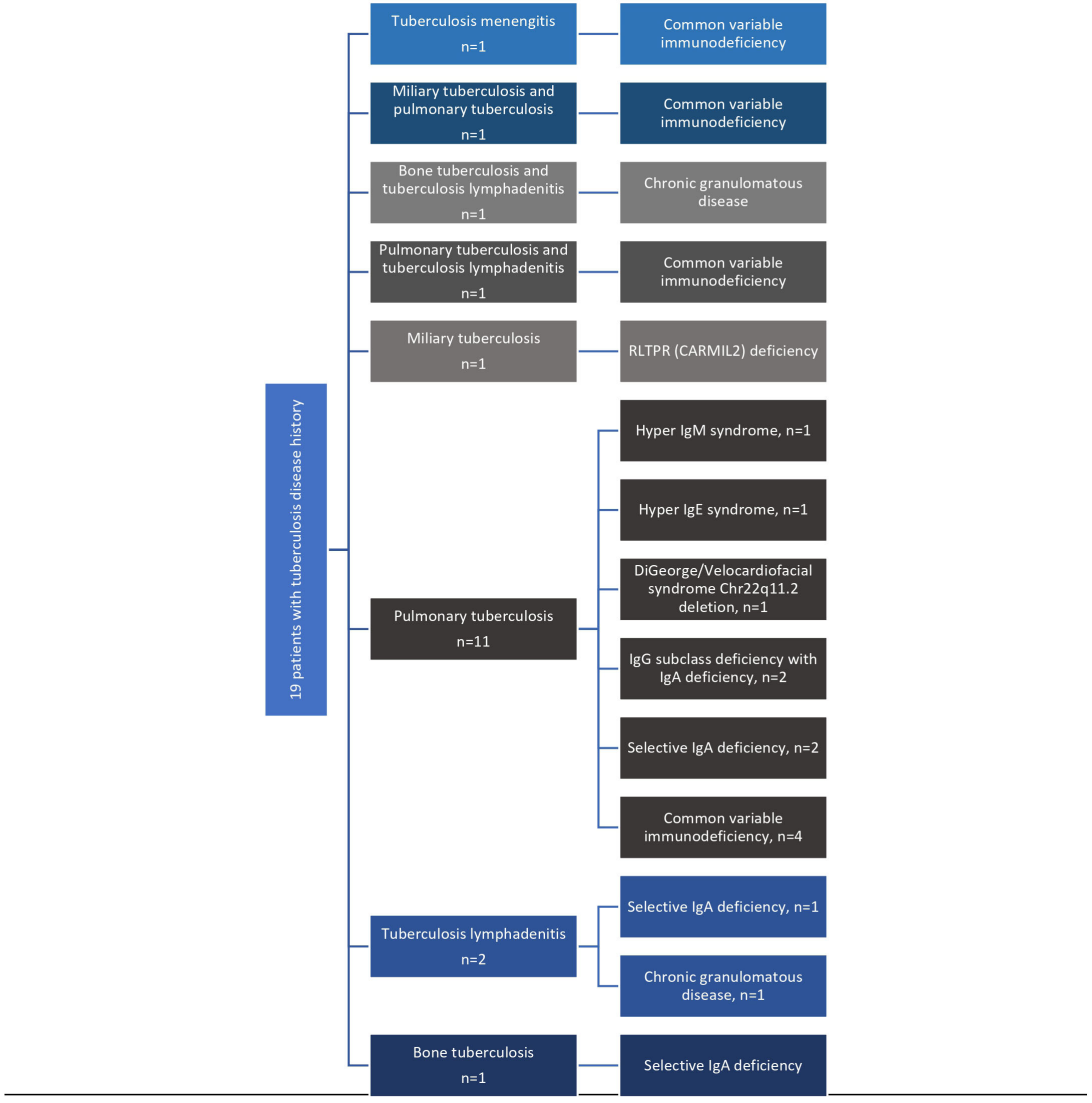
Tissue/organ involvement of previous tuberculosis diseases according to IEI classification.

All patients had a BCG vaccination scar. Six patients (5.1%) reported a family history of tuberculosis, while 19 patients (16.2%) had been previously diagnosed with tuberculosis and had completed antituberculosis treatment. Among these, pulmonary involvement was the most common site, accounting for 59.1% of cases. In addition, three patients had experienced two episodes of tuberculosis. Each episode of tuberculosis was treated according to the recommended treatment durations based on the site of involvement: 12 months for central nervous system tuberculosis, 9 months for skeletal tuberculosis, and 6 months for all other forms. Details of the sites of involvement in prior tuberculosis episodes and the corresponding IEI diagnoses are shown in **Figure 2**.

### TST results and tuberculosis screening outcomes

**Figure 3:**
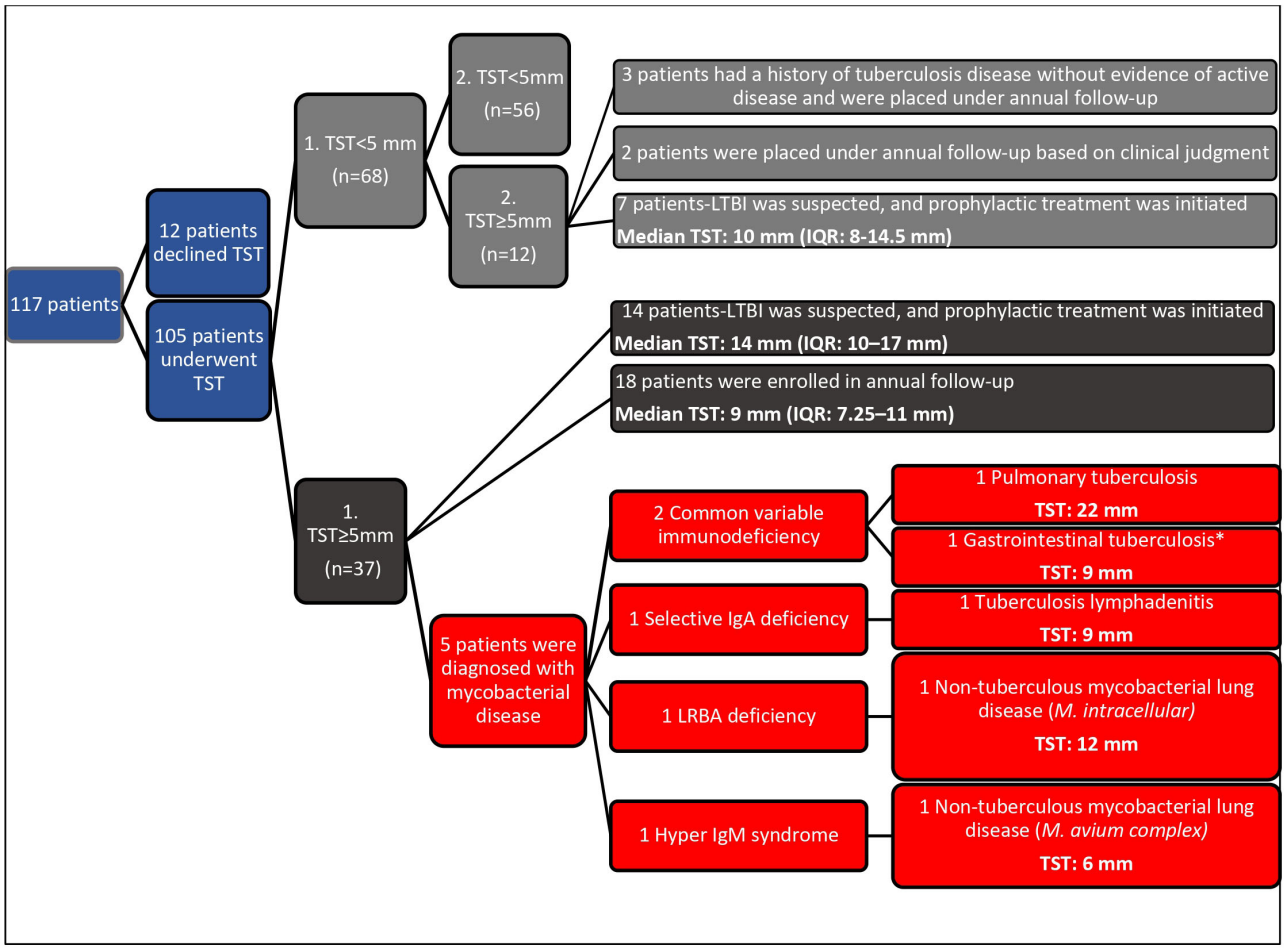
Assessment of active and latent tuberculosis infection. *The patient had a history of pulmonary tuberculosis and was newly diagnosed with gastrointestinal tuberculosis following the screening. TST, Tuberculin skin test; LTBI, Latent tuberculosis infection.

Of the 117 patients with IEI who underwent tuberculosis screening, 105 were assessed with TST. Of these, 49 had an induration of ≥5 mm, including 12 who converted on repeat testing after an initial result of 0–4 mm (**Figure 3**).

Our screening approach identified five patients with newly diagnosed mycobacterial disease. The characteristics of these five patients are presented in **Supplementary Table 2**. Patient 1 died of hemoptysis and pulmonary complications despite receiving antituberculosis treatment. Patient 2 was diagnosed with gastrointestinal tuberculosis based on endoscopic biopsy findings, while sputum cultures were negative for *M. tuberculosis*. Patient 3 presented with palpable axillary lymphadenopathy. According to our clinical protocol, the patient underwent further evaluation, including chest CT, which revealed scattered non-calcified consolidations at the right lung apex. AFB staining and cultures were negative; however, the consolidations did not respond to non-antituberculosis antibiotics. Based on these findings, a clinical consensus was reached that the consolidations were most consistent with pulmonary tuberculosis associated with tuberculous lymphadenitis. A follow-up chest CT scan was scheduled to assess the response to antituberculosis therapy.

A systematic review of thoracic CT scans identified 18 patients with radiological findings consistent with post-tuberculosis sequelae, such as apical fibrotic changes and calcified nodules. Among these, five patients had a history of tuberculosis, two were newly diagnosed with tuberculosis, and one was diagnosed with non-tuberculous mycobacterial infection. One patient did not attend follow-up visits. Three patients received prophylactic therapy. Two patients, both of whom had TST results of 0 mm on two separate occasions, were placed under annual follow-up without treatment following a shared decision between the pulmonology team and the patient. One patient with a TST of 24 mm declined prophylactic therapy. The remaining three patients, with TST results of 6, 8, and 11 mm, were followed annually without treatment based on clinical judgment. Patients with active tuberculosis received standard first-line antituberculosis therapy comprising isoniazid, rifampicin, pyrazinamide, and ethambutol for 2 months, followed by a continuation phase with isoniazid and rifampicin, according to national tuberculosis treatment guidelines. Patients with non-tuberculous mycobacterial lung disease received individualized multidrug regimens based on the isolated species, antimicrobial susceptibility testing, clinical condition, and specialist consultation. The patient with *Mycobacterium intracellulare* lung disease was treated with a linezolid-containing regimen, whereas the patient with *Mycobacterium avium* complex lung disease received macrolide-based multidrug therapy according to current guidelines.

### Evaluation for preventive therapy of latent tuberculosis

Regarding tuberculosis exposure history, five patients had previously received preventive therapy following documented close contact with tuberculosis cases. No additional patients reported similar contact during the follow-up period, and no further preventive therapy was initiated on this basis.

Prophylactic treatment was initiated in 21 patients following specialist consultation. Of the 12 patients who underwent a second TST, seven demonstrated an increase of ≥6 mm from their initial result and subsequently received prophylactic treatment based on a 5-mm threshold established in consultation with pulmonology specialists (**Figure 3**, **Table 3**). In the remaining two patients, a 15-mm threshold was applied in consultation with pulmonology specialists. As their TST increase did not exceed this limit, they were managed with observation alone (**Table 3**). Among the remaining patients (median initial TST = 14 mm, IQR = 10–17 mm), prophylactic treatment indications were evaluated in consultation with pulmonology specialists. Seven patients with an initial TST exceeding 15 mm received prophylactic treatment. In the remaining seven patients, with initial TST values of 10, 14, 10, 9, 10, 8, and 14 mm, a 5-mm threshold was applied following specialist consultation, and prophylactic treatment was initiated accordingly. In all instances, the prophylactic regimen consisted of isoniazid for 9 months. This approach was chosen according to national tuberculosis guidelines, which recommend prolonged prophylaxis for immunocompromised individuals over the standard 6-month regimen recommended for the general population. The clinical details and management decisions for asymptomatic patients with a second TST result of ≥5 mm are presented in **Table 3**. Notably, the prophylaxis decisions were not uniform among patients with booster reactivity. While some patients with second TST measurements between 6 and 14 mm received 9-month isoniazid prophylaxis, others with similar TST responses were managed with annual follow-up alone. These variations reflect individual clinical judgment and the lack of established guidelines for LTBI management in patients with IEI.

**Table 3:** Second tuberculin skin test (TST) evaluation and treatment decisions in patients with inborn errors of immunity (IEI).

Patient	Diagnosis	TST1 (mm)	TST2 (mm)	(ΔTST = TST2 − TST1) (mm)	TST max (mm)	Treatment decision
Patient 1	Common variable immunodeficiency	0	6	6	6	No treatment (annual follow-up)
Patient 2	Hyper IgM syndrome	4	13	9	13	No treatment (annual follow-up)
Patient 3	Selective IgA deficiency	4	15	11	15	9-month isoniazid prophylactic treatment
Patient 4	Common variable immunodeficiency	0	8	8	8	9-month isoniazid prophylactic treatment
Patient 5	Immunodeficiencies affecting cellular and humoral immunity	0	10	10	10	9-month isoniazid prophylactic treatment
Patient 6	Common variable immunodeficiency	0	8	8	8	9-month isoniazid prophylactic treatment
Patient 7	Common variable immunodeficiency	0	15	15	15	9-month isoniazid prophylactic treatment
Patient 8	Common variable immunodeficiency	2	14	12	14	9-month isoniazid prophylactic treatment
Patient 9	Common variable immunodeficiency	0	6	6	6	9-month isoniazid prophylactic treatment
Patient 10	Selective IgA deficiency	4	9	5	9	Previously treated for tuberculosis disease; managed with annual follow-up (no further treatment)
Patient 11	Common variable immunodeficiency	4	9	5	9	Previously treated for tuberculosis disease; managed with annual follow-up (no further treatment)
Patient 12	RLTPR (CARMIL2) deficiency	0	0	0	0	Previously treated for tuberculosis disease; managed with annual follow-up (no further treatment)

TST, tuberculin skin test.

For patients with no documented history of tuberculosis contact who did not meet the criteria for LTBI preventive therapy according to national guidelines, management consisted of routine follow-up with annual re-screening and prompt clinical evaluation if tuberculosis symptoms developed. To date, no cases of tuberculosis or non-tuberculous mycobacterial disease have been detected in this cohort, and monitoring is ongoing.

### TST results in patients with documented tuberculosis and non-tuberculous mycobacterial infections

TST data were available for 12 of 23 patients with previously confirmed or newly diagnosed tuberculosis. The median TST value among 93 patients without tuberculosis was 4 mm (IQR = 0–10.5 mm), whereas the median among patients with tuberculosis was 9 mm (IQR = 1.5–14.25 mm), which remained below the 15-mm threshold generally applied for BCG-vaccinated individuals in the general population. Furthermore, among patients with a documented history of tuberculosis, two patients with selective immunoglobulin A (IgA) deficiency and one patient with RLTPR (CARMIL2) deficiency had TST values of 0 mm on both the initial and second tests. Detailed TST results for patients with previously confirmed and newly diagnosed tuberculosis or non-tuberculous mycobacterial infection are presented in **Table 4**.

**Table 4:** Detailed tuberculin skin test (TST) documentation of tuberculosis and non-tuberculosis mycobacterial infection cases.

Patient	Diagnosis	Initial TST reactivity (mm)	Second TST reactivity (mm)	Symptoms	Type of tuberculosis/mycobacterial infection	Diagnosis time
Patient 1	Common variable immunodeficiency	22		Weight loss, cough, and hemoptysis	Pulmonary tuberculosis	Newly identified infection
Patient 2	Selective IgA deficiency	9		Palpable lymph node in the axillary region	Tuberculosis lymphadenitis	Newly identified infection
Patient 3	LRBA deficiency	12		Asymptomatic, undergoing screening prior to bone marrow transplantation	Non-tuberculous mycobacterial lung disease (*M. intracellulare*)	Newly identified infection
Patient 4	Hyper IgM syndrome	6		Frequent sputum production and recurrent lower respiratory tract infections	Non-tuberculous mycobacterial lung disease (*M. avium* complex)	Newly identified infection
Patient 5	Common variable immunodeficiency	9		Failure to gain weight and intermittent diarrhea	Pulmonary tuberculosis—previous infection Gastrointestinal tuberculosis—newly identified infection	Newly identified infection—recurrent infection
Patient 6	DiGeorge syndrome	20		Asymptomatic	Pulmonary tuberculosis	Previous infection
Patient 7	Selective IgA deficiency	0	0	Asymptomatic	Pulmonary tuberculosis	Previous infection
Patient 8	Selective IgA deficiency	0	0	Asymptomatic	Pulmonary tuberculosis	Previous infection
Patient 9	Selective IgA deficiency	4	9	Asymptomatic	Bone tuberculosis	Previous infection
Patient 10	Common variable immunodeficiency	4	9	Asymptomatic	Miliary tuberculosis and pulmonary tuberculosis	Previous infection
Patient 11	Chronic granulomatous disease	15		Asymptomatic	Bone tuberculosis and tuberculosis lymphadenitis	Previous infection
Patient 12	RLTPR (CARMIL2) deficiency	0	0	Asymptomatic	Miliary tuberculosis	Previous infection

TST, tuberculin skin test.

### T- and B-cell subsets in relation to TST reactivity and tuberculosis history

Among the 69 patients with available data, the T- and B-cell subsets were compared between patients with and without a history of tuberculosis and assessed for correlation with TST reactivity. The only significant finding was an inverse correlation between the percentage of CD8^+^ T cells and TST reactivity (Spearman’s *r* = −0.33, 95%CI = −0.53 to −0.10, *p* = 0.004). No significant differences or correlations were identified in the absolute counts or percentages of other T- and B-cell subsets between patients with and without a history of tuberculosis or in relation to the TST values. Detailed immunophenotypic characteristics of the five patients diagnosed with mycobacterial disease are presented in **Supplementary Table 3**.

## Discussion

To our knowledge, this is the first study to systematically screen for tuberculosis and non-tuberculous mycobacterial infections in patients with IEI using a structured approach. Antibody deficiencies were the predominant IEI subgroup, accounting for 82.1% of the patients. Through this approach, three newly diagnosed tuberculosis cases and two cases of non-tuberculous mycobacterial lung disease were identified. Pulmonary tuberculosis was the most common site of involvement (59.1%), followed by tuberculosis lymphadenitis (18.2%), a distribution not previously described in this population. Of note is that four patients experienced tuberculosis on two separate occasions, highlighting the risk of recurrence among individuals with IEI. Furthermore, the dual-step TST identified booster reactions in nine patients, underscoring its value in LTBI screening and decisions regarding preventive therapy. These findings highlight the need to establish tailored tuberculosis screening protocols for patients with IEI, a population for whom no established LTBI screening guidelines currently exist.

CVID is widely recognized for its association with recurrent infections, predominantly caused by encapsulated bacteria; however, its relationship with tuberculosis has not been fully elucidated. Reports of tuberculosis in patients with CVID are scarce, with only a few case reports in the literature (25, 26). Of the 23 patients diagnosed with tuberculosis, eight had CVID without an identifiable cellular immune defect predisposing them to tuberculosis. These findings highlight the notable occurrence of tuberculosis among patients with CVID, particularly in tuberculosis-endemic regions. Tuberculosis remains endemic in Türkiye, with an estimated incidence of 11.0 per 100,000 population in 2023 as reported by the WHO. The frequency of tuberculosis among patients with CVID in our cohort underscores the need for heightened clinical vigilance and systematic screening in such settings (7). In one patient with CVID receiving routine intravenous immunoglobulin (IVIG) therapy, *M. tuberculosis* was detected in biopsies from a lesion identified on routine annual endoscopy, a finding that is particularly noteworthy. To our knowledge, this has not been previously reported in patients with CVID. Although tuberculosis at atypical sites has been documented in immunosuppressed individuals, such as those with neuroendocrine tumors or HIV, this case suggests that immune impairment in IEI may predispose patients to tuberculosis involvement at uncommon locations (27, 28). This highlights the need for awareness of atypical tuberculosis presentations in this population.

T-cell function can be assessed *in vivo* using the TST, with a conventional threshold of ≥5 mm indicating preserved T-cell activity (29). In our study—where antibody deficiencies constituted the majority of diagnoses—56 patients had TST reactivity of 0–4 mm on both the initial and second tests, despite prior BCG vaccination, which can elevate the TST readings in the general population. This raises an important question regarding the reliability of TST in assessing T-cell function in this population. Although our cohort consisted primarily of patients with antibody deficiencies, impaired cellular immunity has been increasingly recognized in several antibody deficiency disorders, particularly CVID (30). Abnormalities in T-cell activation, cytokine production, and immune regulation have been reported in these patients and may contribute to reduced TST responsiveness (30). Therefore, the reduced TST responses observed in our cohort may not solely reflect limitations of the TST itself but may also indicate underlying cellular immune dysregulation associated with the primary disease. In light of this, interpreting the TST results in conjunction with *in vitro* T-cell function assays may be preferable, as TST alone may underestimate true immune competence in this population. Furthermore, among patients with active tuberculosis, where a TST induration of ≥15 mm would typically be expected, two patients had an induration of only 9 mm, further complicating the interpretation of the TST results in these patients. These observations suggest that further research is warranted to evaluate the utility and limitations of TST in patients with IEI, particularly in combination with other measures of immune function. A further challenge in interpreting TST results is the considerable variability observed among patients with similar IEI diagnoses. This heterogeneity indicates that TST responsiveness may be affected not only by diagnostic category but also by individual variations in immune dysfunction, disease severity, treatment exposure, and prior mycobacterial exposure. Consequently, the TST results should be interpreted with caution and not used in isolation when assessing tuberculosis risk in patients with IEI. Instead, the TST should be regarded as one element within a comprehensive evaluation incorporating clinical, radiological, epidemiological, and microbiological data.

The approach to LTBI prophylaxis in patients with IEI is not well established, with some clinicians managing them similarly to patients with HIV and others applying general population thresholds. In the absence of established international or national recommendations for LTBI prophylaxis in patients with IEI, we describe our tuberculosis screening strategy for the first time, comprising routine assessment of tuberculosis-related symptoms, TST, and lung imaging, which may serve as a valuable guide for clinicians. This variability in clinical practice was apparent in our study, in which 21 patients received LTBI prophylaxis: seven identified through booster reactivity, seven exceeding the 15-mm threshold, and seven with TST values between 5 and 14 mm. All patients receiving prophylaxis were prescribed a 9-month course of isoniazid, as recommended for patients with HIV. The TST responses in patients with IEI were often lower than expected, with a median induration of 9 mm among those with a history of tuberculosis. Among the three newly diagnosed patients, one had a TST of 22 mm, while two had values of only 9 mm, reinforcing that even active tuberculosis may not elicit a robust TST response. A strict 15-mm threshold would have missed four of the five confirmed mycobacterial disease cases. Therefore, we recommend a comprehensive assessment incorporating TST reactivity (≥5 mm) alongside additional risk factors to minimize the risk of underdiagnosis in this population.

In our study, four patients experienced two separate episodes of tuberculosis, despite completing adequate treatment for their initial episode. Among them, one patient had CGD, while the remaining three had CVID. Among the three patients with CGD in this cohort, one had previously received prophylactic tuberculosis treatment and has not developed tuberculosis to date. Conversely, the other two developed tuberculosis, one of whom experienced two episodes. CGD is a primary immunodeficiency characterized by defective phagocytic function, predisposing affected individuals to recurrent bacterial and fungal infections and tuberculosis. Consequently, tuberculosis screening is recommended for all patients with CGD (31).

Although this study provided valuable insights for clinicians, certain limitations should be acknowledged. Firstly, it is a single-center study conducted in Istanbul. We recommend that tuberculosis screening be implemented in larger cohorts of patients with IEI in countries with low tuberculosis incidence (0–9.9 per 100,000 population), as the prevalence of tuberculosis in this population may be higher than generally assumed. Another limitation is the variability in clinical decision-making resulting from the involvement of multiple pulmonologists, which precluded a standardized approach to LTBI prophylaxis. Nonetheless, this variability highlights a key finding of this study: the absence of a unified strategy for the management of LTBI in patients with IEI. To our knowledge, this is the first study to document the heterogeneity of physician approaches to LTBI prophylaxis in this population, and this variability may have influenced individual clinical decisions. A further limitation is the absence of systematic IGRA testing. Although IGRA could have provided valuable supplementary information in our cohort, which was universally BCG-vaccinated, routine access to IGRA was limited during the study period owing to availability and cost constraints. As a result, a direct comparison between TST and IGRA was not feasible. Future studies incorporating both diagnostic modalities may help refine the tuberculosis screening strategies in patients with IEI.

In conclusion, this study represents the first comprehensive evaluation of tuberculosis screening and TST assessment in patients with IEI. It also systematically documented sites of tuberculosis involvement in this population, providing new insights into disease distribution in patients with IEI. Among the BCG-vaccinated patients with IEI who had tuberculosis, TST reactivity did not reach the 15-mm threshold typically expected in the general population. Our findings suggest that conventional TST interpretation may not be appropriate for all patients with IEI and highlight the need for further investigation in larger multicenter cohorts. In the absence of standardized tuberculosis screening protocols for patients with IEI, the systematic approach used at our center may serve as a practical framework for tuberculosis screening in this population. Further validation in larger multicenter studies is warranted.

## Data Availability

The raw data supporting the conclusions of this article will be made available by the authors, without undue reservation.

## References

[1] Allenspach E Torgerson TR . Autoimmunity and primary immunodeficiency disorders. J ClinImmunol. (2016) 36:57–67. doi: 10.1007/s10875-016-0294-1 27210535

[2] Milner JD Holland SM . The cup runneth over: lessons from the ever-expanding pool of primary immunodeficiency diseases. Nat Rev Immunol. (2013) 13:635–48. doi: 10.1038/nri3493 23887241

[3] Tangye SG Al-Herz W Bousfiha A Cunningham-Rundles C Franco JL Holland SM . Human inborn errors of immunity: 2022 update on the classification from the International Union of Immunological Societies Expert Committee. J ClinImmunol. (2022) 42:1473–507. doi: 10.1007/s10875-022-01289-3 35748970 PMC9244088

[4] Bustamante J Boisson-Dupuis S Abel L Casanova JL . Mendelian susceptibility to mycobacterial disease: genetic, immunological, and clinical features of inborn errors of IFN-gamma immunity. Semin Immunol. (2014) 26:454–70. doi: 10.1016/j.smim.2014.09.008 25453225 PMC4357480

[5] Marciano BE Huang CY Joshi G Rezaei N Carvalho BC Allwood Z . BCG vaccination in patients with severe combined immunodeficiency: complications, risks, and vaccination policies. J Allergy Clin Immunol. (2014) 133:1134–41. doi: 10.1016/j.jaci.2014.02.028 24679470 PMC4015464

[6] Roos D de Boer M . Molecular diagnosis of chronic granulomatous disease. Clin Exp Immunol. (2014) 175:139–49. doi: 10.1111/cei.12202 24016250 PMC3892405

[7] World Health Organization. Global tuberculosis report 2024. (2024). Geneva: World Health Organization.

[8] Deng G Zhang P Lu H . Challenges in the screening and treatment of latent multidrug-resistant tuberculosis infection. Drug Discov Ther. (2022) 16:52–4. doi: 10.5582/ddt.2022.01029 35466125

[9] Lardizabal AA Reichman LB . Diagnosis of latent tuberculosis infection. Microbiol Spectr. (2017) 5. doi: 10.1128/microbiolspec.TNMI7-0019-2016 28185619 PMC11687433

[10] Pahal P Pollard EJ Sharma S . PPD skin test. In: Statpearls. Treasure Island (FL): StatPearls Publishing (2024). 32310497

[11] Ahmet Gorkem Er AIS Asli Sule T Aylin Babalik Aysegül Yildirim Derya Oztomurcuk . Tuberculosis Diagnosis and Treatment Guide. Kara F , editor. Turkey: General Directorate of Public Health of Turkey (2019).

[12] Singh JA Furst DE Bharat A Curtis JR Kavanaugh AF Kremer JM . 2012 update of the 2008 American College of Rheumatology recommendations for the use of disease-modifying antirheumatic drugs and biologic agents in the treatment of rheumatoid arthritis. Arthritis Care Res (Hoboken). (2012) 64:625–39. doi: 10.1002/acr.21641 22473917 PMC4081542

[13] Teixeira EG Kritski A Ruffino-Netto A Steffen R Lapa ESJR Belo M . Two-step tuberculin skin test and booster phenomenon prevalence among Brazilian medical students. Int J Tuberc Lung Dis. (2008) 12:1407–13. Available online at: https://www.ncbi.nlm.nih.gov/pubmed/19017450 (Accessed July 20, 2026). 19017450

[14] Salles CG Ruffino-Netto A Lapa-e-Silva JR Kritski AL Cailleaux-Cesar M Queiroz-Mello FC . The presence of a booster phenomenon among contacts of active pulmonary tuberculosis cases: a retrospective cohort. BMC Public Health. (2007) 7:38. doi: 10.1186/1471-2458-7-38 17371600 PMC1839086

[15] Erratum: Treatment of drug-resistant tuberculosis. An official ATS/CDC/ERS/IDSA clinical practice guideline. Am J Respir Crit Care Med. (2020) 201:500–1. doi: 10.1164/rccm.v201erratum2 32057258 PMC7049913

[16] Sterling TR Njie G Zenner D Cohn DL Reves R Ahmed A . Guidelines for the treatment of latent tuberculosis infection: recommendations from the National Tuberculosis Controllers Association and CDC, 2020. MMWR Recomm Rep. (2020) 69:1–11. doi: 10.15585/mmwr.rr6901a1 32053584 PMC7041302

[17] Ameratunga R Longhurst H Steele R Woon ST . Comparison of diagnostic criteria for common variable immunodeficiency disorders (CVID) in the New Zealand CVID cohort study. Clin Rev Allergy Immunol. (2021) 61:236–44. doi: 10.1007/s12016-021-08860-7 34236581

[18] Bousfiha AA Jeddane L Moundir A Poli MC Aksentijevich I Cunningham-Rundles C . The 2024 update of IUIS phenotypic classification of human inborn errors of immunity. J Hum Immun. (2025) 1:e20250002. doi: 10.70962/jhi.20250002 41608113 PMC12829316

[19] Poli MC Aksentijevich I Bousfiha AA Cunningham-Rundles C Hambleton S Klein C . Human inborn errors of immunity: 2024 update on the classification from the International Union of Immunological Societies Expert Committee. J Hum Immun. (2025) 1:e20250003. doi: 10.70962/jhi.20250003 41608114 PMC12829761

[20] Seidel MG Kindle G Gathmann B Quinti I Buckland M van Montfrans J . The European society for immunodeficiencies (ESID) registry working definitions for the clinical diagnosis of inborn errors of immunity. J Allergy Clin Immunol Pract. (2019) 7:1763–70. doi: 10.1016/j.jaip.2019.02.004 30776527

[21] Baris S Abolhassani H Massaad MJ Al-Nesf M Chavoshzadeh Z Keles S . The Middle East and North Africa diagnosis and management guidelines for inborn errors of immunity. J Allergy Clin Immunol Pract. (2023) 11:158–80:e111. doi: 10.1016/j.jaip.2022.10.003 36265766

[22] Chappity P Mamidi D Pradhan S . Diagnostic high grade tuberculin sensitivity. IDCases. (2021) 24:e01085. doi: 10.1016/j.idcr.2021.e01085 33889486 PMC8050023

[23] Kim HY Song KS Goo JM Lee JS Lee KS Lim TH . Thoracic sequelae and complications of tuberculosis. Radiographics. (2001) 21:839–58. doi: 10.1148/radiographics.21.4.g01jl06839. discussion 859-860. 11452057

[24] Palmer PE . Pulmonary tuberculosis--usual and unusual radiographic presentations. Semin Roentgenol. (1979) 14:204–43. doi: 10.1016/0037-198x(79)90007-5 472765

[25] Balwani MR Kute VB Shah PR Wakhare P Trivedi HL . Secondary renal amyloidosis in a patient of pulmonary tuberculosis and common variable immunodeficiency. J Nephropharmacol. (2015) 4:69–71. Available online at: https://www.ncbi.nlm.nih.gov/pubmed/28197481 (Accessed July 20, 2026). 28197481 PMC5297488

[26] Nepesov S Aygun FD Firtina S Cokugras H Camcioglu Y . Clinical and immunological features of 44 common variable immunodeficiency patients: the experience of a single center in Turkey. Allergol Immunopathol (Madr). (2020) 48:675–85. doi: 10.1016/j.aller.2019.12.008 32299645

[27] Brabo EP Viana M Caroli-Bottino A Pannain VLN Eiras A Moraes AB . Colonic tuberculosis presenting as intestinal subocclusion in a patient with neuroendocrine tumor: a case report. AME Case Rep. (2021) 5:36. doi: 10.21037/acr-21-28 34805755 PMC8572667

[28] Li C Zhang Q Wen Y . Intestinal tuberculosis presented as spindle cell pseudotumor in a HIV-positive case. Rev Esp Enferm Dig. (2023) 115:723–4. doi: 10.17235/reed.2023.9533/2023 36866839

[29] Blatt SP Hendrix CW Butzin CA Freeman TM Ward WW Hensley RE . Delayed-type hypersensitivity skin testing predicts progression to AIDS in HIV-infected patients. Ann Intern Med. (1993) 119:177–84. doi: 10.7326/0003-4819-119-3-199308010-00001 8100691

[30] Azizi G Rezaei N Kiaee F Tavakolinia N Yazdani R Mirshafiey A . T-cell abnormalities in common variable immunodeficiency. J Investig Allergol Clin Immunol. (2016) 26:233–43. doi: 10.18176/jiaci.0069 27374799

[31] Vignesh P Sil A Aggarwal R Laha W Mondal S Dhaliwal M . Tuberculosis and Bacillus Calmette-Guerin disease in patients with chronic granulomatous disease: an experience from a tertiary care center in North India. J ClinImmunol. (2023) 43:2049–61. doi: 10.1007/s10875-023-01581-w 37721651

