# Supplementary material for: Tuberculosis screening in adult patients with inborn errors of immunity: a single-center cohort study
Source: Front Immunol. 2026 Jul 24;17:1888729. doi: 10.3389/fimmu.2026.1888729 (PMC13447173; doi:10.3389/fimmu.2026.1888729)
Supplement: Supplementary file 1 [file Table1.docx]

**Supplementary Table 1.** Molecular diagnoses identified in the 20 genetically confirmed patients.

| **Gene** | **Molecular diagnosis** | **No. of patients** |
| --- | --- | --- |
| *CTLA4* | CTLA4 deficiency | 3 |
| *BTK* | X-linked agammaglobulinemia | 3 |
| *CYBB* | Chronic granulomatous disease | 3 |
| *PIK3CD* | Activated PI3Kδ syndrome (APDS) | 2 |
| *LRBA* | LRBA deficiency | 1 |
| *RLTPR (CARMIL2)* | CARMIL2 deficiency | 1 |
| *GATA2* | GATA2 deficiency | 1 |
| *AICDA* | AID deficiency (Hyper-IgM syndrome type 2) | 1 |
| *22q11.2 deletion* | DiGeorge syndrome (22q11.2 deletion syndrome) | 1 |
| *PGM3* | PGM3 deficiency | 1 |
| *STAT3* | Autosomal dominant hyper-IgE syndrome (STAT3 deficiency) | 1 |
| *ATM* | Ataxia-telangiectasia | 1 |
| *POLE1* | POLE1 deficiency | 1 |
| *DOCK8* | DOCK8 deficiency | 1 |
| **Total** |  | **20** |

AID, activation-induced cytidine deaminase; APDS, activated PI3Kδ syndrome; ATM, ataxia telangiectasia mutated; BTK, Bruton tyrosine kinase; CARMIL2, capping protein regulator and myosin 1 linker 2; CTLA4, cytotoxic T-lymphocyte-associated protein 4; CYBB, cytochrome b-245 beta chain; DOCK8, dedicator of cytokinesis 8; GATA2, GATA binding protein 2; LRBA, lipopolysaccharide-responsive beige-like anchor protein; PGM3, phosphoglucomutase 3; PIK3CD, phosphatidylinositol-4,5-bisphosphate 3-kinase catalytic subunit delta; POLE1, DNA polymerase epsilon subunit 1; RLTPR, RGD motif leucine-rich repeats tropomodulin domain and proline-rich containing; STAT3, signal transducer and activator of transcription 3.
