# Supplementary material for: Tuberculosis screening in adult patients with inborn errors of immunity: a single-center cohort study
Source: Front Immunol. 2026 Jul 24;17:1888729. doi: 10.3389/fimmu.2026.1888729 (PMC13447173; doi:10.3389/fimmu.2026.1888729)
Supplement: Supplementary file 2 [file Table2.docx]

**Supplementary Table 2.** Characteristics of IEI Patients Diagnosed with Mycobacterial Infections Through Our Diagnostic Approach

| Patients | **Diagnosis** | **Symptoms and signs** | **History of contact with a tuberculosis patient** | **TST reactivity** | **Thorax computed tomography findings** | **Tuberculosis culture result** | **Type of mycobacterial disease** |
| --- | --- | --- | --- | --- | --- | --- | --- |
| **Patient 1** | Common variable immunodeficiency | Weight loss, cough, and haemoptysis | No history of contact with tuberculosis | 22 mm | Multilobed, thin-walled cavitary lesions. Calcified sequelae in the lung apices | *Mycobacterium tuberculosis* | Pulmonary tuberculosis |
| **Patient 2** | Common variable immunodeficiency | Failure to gain weight and intermittent diarrhoea | No history of contact with tuberculosis | 9 mm | Calcified sequelae in the lung apices and bronchiectasis | *Mycobacterium tuberculosis* | Gastrointestinal tuberculosis |
| **Patient 3** | Selective IgA deficiency | Palpable lymph node in the axillary region | No history of contact with tuberculosis | 9 mm | Scattered non-calcified consolidation in the right lung apex (sputum AFB and culture negative) | *Mycobacterium tuberculosis* | Tuberculosis lymphadenitis |
| **Patient 4** | LRBA deficiency | Asymptomatic, undergoing screening prior to bone marrow transplantation | No history of contact with tuberculosis | 12 mm | Scattered areas of ground-glass opacity, bronchiectasis changes, and focal calcified consolidations | *M. intracellular* | Non-tuberculous mycobacterial lung disease |
| **Patient 5** | Hyper IgM syndrome | Frequent sputum production and recurrent lower respiratory tract infections | No history of contact with tuberculosis | 6 mm | Multilobed, widespread cystic, sacculated, and varicose bronchiectasis. Calcified sequelae in the lung apices | *M. avium complex* | Non-tuberculous mycobacterial lung disease |

TST: Tuberculin skin test
