# Supplementary material for: Tuberculosis screening in adult patients with inborn errors of immunity: a single-center cohort study
Source: Front Immunol. 2026 Jul 24;17:1888729. doi: 10.3389/fimmu.2026.1888729 (PMC13447173; doi:10.3389/fimmu.2026.1888729)
Supplement: Supplementary file 3 [file Table3.docx]

**Supplementary Table 3.** Genetic and Immunological Characteristics of Patients with Mycobacterial Disease

| Patients | **Diagnosis** | **Genetic defect (variant)** | **CD4 count (/µL)** | **CD8 count (/µL)** | **CD4/CD8 ratio** | **CD19 B cells (%)** | **Ig replacement therapy** | **Immunomodulatory treatment** |
| --- | --- | --- | --- | --- | --- | --- | --- | --- |
| **Patient 1** | Common variable immunodeficiency | No pathogenic variant detected | 320 | 800 | 0.4 | 4 | Yes | No |
| **Patient 2** | Common variable immunodeficiency | No pathogenic variant detected | 350 | 714 | 0.49 | 8.44 | Yes | No |
| **Patient 3** | Selective IgA deficiency | No pathogenic variant detected | 510 | 386 | 1.32 | 9 | No | No |
| **Patient 4** | LRBA deficiency | Compound heterozygous LRBA variants: NM_001364905.1(LRBA):c.1963C>T (p.Arg655Ter) and NM_001364905.1(LRBA):c.3156delT (p.Asp1053Thrfs*2) | 240 | 399 | 0.6 | 0.07 | Yes | Abatacept |
| **Patient 5** | Hyper IgM syndrome | No pathogenic variant detected | 302 | 890 | 0.33 | 7 | Yes | No |
| CD4, cluster of differentiation  Reference ranges used by our institutional flow cytometry laboratory:  CD4+ T cells, 529–1718 cells/µL; CD8+ T cells, 213–1120 cells/µL;  CD4/CD8 ratio, 0.9–3.6; CD19+ B cells, 5.17–19.23%. | | | | | | | | |
